# Draft Genome Sequence of the Yeast *Ogataea degrootiae* Strain UCD465, Isolated from Soil in Ireland

**DOI:** 10.1128/MRA.00736-21

**Published:** 2021-09-30

**Authors:** Eoin Ó Cinnéide, Max Jones, Elijah Bahate, Elizabeth Boyd, Rebeca Clavero, Harry Doherty, Izabela Drozdz, Maja Dumana, Cristina Gonzales, Jane Kennedy, Maria Khan, Shane Maher, Clare McGurk, Katherine Moreau, Ruby Neville, Jade Norton, Tadhg Ó Cróinín, Elizabeth O’Gorman, Juliana C. Olliff, Rhys Orimaco, Ellen Quinn, Sarah Webb Kennedy, Sean A. Bergin, Kevin P. Byrne, Kenneth H. Wolfe, Geraldine Butler

**Affiliations:** a School of Biomolecular and Biomedical Science, Conway Institute, University College Dublin, Dublin, Ireland; b School of Medicine, Conway Institute, University College Dublin, Dublin, Ireland; Vanderbilt University

## Abstract

Ogataea degrootiae is an ascomycete yeast that was first isolated in the Netherlands in 2017. It is a member of the Pichiaceae clade. Here, we present the genome sequence of O. degrootiae UCD465, which was isolated from soil in Ireland. This genome is 14.6 Mb and haploid.

## ANNOUNCEMENT

*Ogataea* is an ascomycete yeast genus belonging to the family Pichiaceae in the subphylum Saccharomycotina (1). There are more than 30 species of *Ogataea* (2). The type strain of Ogataea degrootiae, CBS 15033, was isolated in 2017 from garden soil in the Netherlands (3). We identified another isolate, O. degrootiae UCD465, from soil collected in County Sligo, Ireland (coordinates: 54.227545, −9.031879), by two passages of soil material in 9 ml liquid yeast extract-peptone-dextrose (YPD) containing chloramphenicol (30 μg/ml) and ampicillin (100 μg/ml) and culture on YPD plates at room temperature, similar to the procedure described previously (4). The species was identified from single colonies by PCR amplification and Sanger sequencing of the internal transcribed spacer (ITS) and D1/D2 regions of its ribosomal DNA locus, both of which were identical to those of O. degrootiae CBS 15033 (3) (GenBank accession numbers NR_168172.1 and NG_068257.1, respectively).

Total genomic DNA was extracted from a YPD culture using phenol-chloroform-isoamyl alcohol. DNA was precipitated with isopropanol and ammonium acetate, washed twice in 70% ethanol, and dissolved in 100 μl Tris-EDTA (TE) buffer. Libraries were generated and sequenced by BGI Tech Solutions Co. (Hong Kong). A total of 1 μg genomic DNA was fragmented and size selected using a Covaris ultrasonicator, purified with an AxyPrep Mag PCR clean-up kit, and end repaired, and A-tails were added by using an A-tailing mix and incubating the mixture at 37°C for 30 min. Illumina adapters were ligated by incubation at 16°C for 16 h. Approximately 150 bases were sequenced from each end of ∼800-bp inserts with an Illumina HiSeq 4000 instrument, yielding 7.1 million read pairs.

Low-quality reads and adapter sequences were removed using Skewer v.0.2.2 (5) with default parameters. The genome was assembled using SPAdes v.3.11.1 (6) with the careful parameter. Based on coverage-versus-length plot analysis (7), scaffolds with less than 10× coverage or 0.5-kb length were removed, leaving 410 scaffolds. The assembly was analyzed using QUAST v.4.6.1 (8). The total genome size is 14.6 Mb, which is larger than the ∼9-Mb genomes of *Ogataea* species Ogataea polymorpha and Ogataea parapolymorpha but is similar to the assemblies of closer relatives Ogataea methanolica and Ogataea trehalophila (1, 3). The *N*_50_ value is 95,261 bp, the *L*_50_ value is 49 contigs, and the G+C content is 36.2%. The largest contig is 343,079 bp, and the average coverage is 60×. Using BUSCO v.5.2.2 (9), genome completeness was estimated at 94.0% (compared to the Ascomycota lineage data set).

To examine ploidy and heterozygosity, heterozygous single-nucleotide polymorphisms (SNPs) were identified by aligning the trimmed reads to the assembled genome using BWA v.0.7.12-r1039 (10) (parameters bwa mem -M -Y -t 2 -R, followed by samtools view -S -b, samtools sort, and samtools flagstat with default parameters and then picard-tools MarkDuplicates and picard-tools BuildBamIndex with VALIDATION_STRINGENCY=LENIENT). Variants were called with HaplotypeCaller from GATK v.4.0.1.2 (11) with default parameters. Variants were filtered using GATK VariantFiltration with parameters -cluster-size 5 -cluster-window-size 20, followed by -genotype-filter-expression GQ < 20 -genotype-filter-expression DP < 10. Only 2,547 heterozygous sites were identified, suggesting that O. degrootiae UCD465 has a haploid genome.

We found mating-type loci with both *MAT***a** and *MAT*α genotypes, on different contigs, i.e., genes *MAT***a***1* and *MAT***a***2* on JAHDIT010000230.1 and genes *MAT*α1 and *MAT*α2 on JAHDIT010000187.1. It is thus likely that O. degrootiae is homothallic and switches mating types by inversion of a section of its genome (12).

### Data availability.

This whole-genome shotgun project was deposited in DDBJ/ENA/GenBank (accession number JAHDIT000000000). The version described in this paper is version JAHDIT010000000. The raw reads were deposited in the SRA (accession number SRR14551582). These data are also available under BioProject number PRJNA730000. The ITS sequence is available under accession number MZ191776 and the D1/D2 sequence under accession number MZ191775.
